# Evaluation of Clinical Remission in Best-Performing Severe Asthmatic Patients Treated for Three Years with Mepolizumab

**DOI:** 10.3390/biomedicines12050960

**Published:** 2024-04-26

**Authors:** Diego Bagnasco, Benedetta Bondi, Marco Caminati, Stefania Nicola, Laura Pini, Manlio Milanese, Luisa Brussino, Gianenrico Senna, Giorgio Walter Canonica, Fulvio Braido

**Affiliations:** 1 Respiratory and Allergy Clinic, IRCCS Ospedale Policlinico San Martino, 16132 Genoa, Italy; 2 Department of Internal Medicine (DIMI), University of Genoa, 16132 Genoa, Italy; 3 Department of Medicine, University of Verona, 37129 Verona, Italy; 4 SCDU Immunology and Allergology, AO Ordine Mauriziano, 10128 Turin, Italy; 5 Respiratory Medicine Unit, ASST—Spedali Civili di Brescia, 25123 Brescia, Italy; 6 Department of Respiratory Diseases, S. Corona Hospital, ASL2, 17027 Pietra Ligure, Italy; 7 Department of Biomedical Sciences, Humanitas University, Via Rita Levi Montalcini 4, Pieve Emanuele, 20090 Milan, Italy

**Keywords:** asthma, complete remission, mepolizumab, partial remission, severe asthma

## Abstract

Background: In its severe form, where possible, asthma is treated using biological drugs in order to reduce, as much as possible, the use of systemic steroids. Mepolizumab is effective for severe asthma based on key outcomes such as exacerbation and steroid dependence. Its efficacy in terms of the criteria for clinical remission in the short and long term has become of interest. Objective: We aimed to evaluate the effect of mepolizumab in the achievement of clinical remission after 3 years of administration. Methods: In this study, 71 patients who continued mepolizumab for 3 years were assessed for clinical remission according to six different published sets of remission criteria. Results: According to the criteria, 39–52% of patients experienced complete remission in the first year, increasing to 51–73% at 3 years. By classifying patients according to partial and complete remission criteria, proposed by the SANI, we observe 22% of patients in partial remission at one year, achieving complete remission after three years. The baseline factors associated with earlier remission were a higher FEV1, if we consider classifications requiring an FEV1 ≥ 80%, a low OCS dose, and low FeNO levels, in the patients requiring FEV1 stabilization. Conclusions: Clinical remission is possible for patients treated with mepolizumab. The observations at three years compared with the first year indicated that the factors negatively affecting remission delayed rather than prevented it. Earlier treatment could increase the chances of remission.

## 1. Introduction

Although asthma is usually treatable with an adequate inhaled steroid with or without a second controller such as long-acting beta 2 agonists, about 5–10% of patients have a severe form [1]. The widely used definition of severe uncontrolled asthma is that which was developed from the American Thoracic Society (ATS)/European Respiratory Society (ERS) guidelines [2], later incorporated into the Global Initiative for Asthma guidelines. In these guidelines, “severe” is defined as poor symptom control despite a maximum dosage of inhaled therapy, requiring a systemic corticosteroid to reduce the exacerbation risk. To achieve disease control in these cases, biologic drugs that interact with specific inflammatory pathways are increasingly being used. Among the goals for these patients are reduced exacerbations and reduced systemic corticosteroid use, along with disease control and a decreased impact on their quality of life.

Clinical trials often rely on individual parameters taken singly as endpoints in evaluating the drug’s effectiveness. An increasingly common outcome in trials is clinical disease remission. No single definition has been published that expresses this concept in severe asthma, but criteria are available that consider several parameters at once in defining remission. Among these are exacerbation, steroid use, and especially, the ability to discontinue systemic steroid therapy, disease control, and respiratory function. The various proposed definitions specifically differ in the respiratory function, with an FEV1 ≥ 80% of the predicted value proposed by some, while others suggest the more generic concept of stabilization. The main objective of this work is to analyze clinical remission rates and differences in remission rates according to the different criteria in patients treated for 3 years with the anti-interleukin-5 (IL-5) biological drug mepolizumab, which is an humanized IgG(1) monoclonal antibody able to bind IL-5, preventing the association of the cytokine with its receptor, used for severe hypereosinophilic asthma. Secondary endpoints were the effectiveness for exacerbations, oral corticosteroid (OCS) sparing, and respiratory function. We also conducted a comparison with previously published observations regarding attaining remission with mepolizumab.

## 2. Methods

### 2.1. Study Design and Patient Population

This retrospective, real-world study included patients with severe asthma according to the 2014 ATS/ERS criteria, enrolled from five Italian national referral centers (Genoa, Verona, Turin, Brescia, and Pietra Ligure). Patients who benefited from the treatment and continued it for at least 36 months were analyzed. We analyze the data of about 60% of the patients featured in our publication [3], particularly those of the groups designated to signing the informed consent for this analysis, resulting in analogue baseline characteristics (sex, age, stage of therapy, eosinophils levels, and lung function). In this manuscript, we aim to focus attention towards the primary endpoint of the new concept of clinical remission while also examining duration and persistence over a period of 3 years into remission, according to different definitions. Additionally, we are also the first to utilize the Italian criteria defined by SANI to study partial and complete remission (not long term efficacy, as this was already studied in the previously mentioned manuscript). The only inclusion criterion was the ability to prescribe mepolizumab (GlaxoSmithKline Manufacturing S.p.A, San Polo di Torrile, Parma, Italia), according to the Italian regulatory agency therapeutic plan (severe uncontrolled asthma according to ATS/ERS guidelines, STEP 4/5 GINA therapy, at least 2 exacerbations requiring OCS in the previous year or steroid dependance, a blood eosinophil count of at least 150 cells/µL at the beginning of therapy, and at least 300 cells/µL in the previous year) [4]. No patients were excluded. Only patients with all available data, for 3 consecutive years, on what is required to define them as being in remission or not (FEV1, ACT, exacerbations, and OCS use) were considered. At baseline, all patients met the criteria for uncontrolled severe asthma as well as the indications listed by the Italian regulatory authorities for prescribing mepolizumab in asthma [4]. All patients signed an informed consent to be part of the observation study, and the project received favorable opinion by the Genoa ethics committee (year 2017, ID 3663).

### 2.2. Observation Time

The observation time points were at 12 months, 24 months, and 36 months. Exacerbations, as well as OCS use, always refer to the 12 months between one observation point and the previous one; therefore, those related to the baseline refer to the number/dose, recorded in the 12 months prior to the beginning of therapy, the first year between baseline and the first 12 months, the second year between 12 and 24 months, and the third year between 24 and 36 months. Where specified, to analyze the rate of patients maintaining r the emission for all the observation periods, an assessment was also made between exacerbations and the OCS dose relative to the comparison between the beginning of therapy (baseline) and 36 months (third year).

### 2.3. Remission Definition

Definitions of disease remission that were applied and compared were taken from the REal worlD Effectiveness and Safety of Mepolizumab (REDES) study [5], Thomas et al. [6] (given here as “Thomas” in brief), the Severe Asthma Network in Italy (SANI)’s partial and completed clinical remission language [7], the one by Lommatzsch [8] et al. (given here as “Lommatzsch” in brief), and Menzies-Gow et al. [9] (given here as “Menzies-Gow” in brief).

The REDES definition is based on four components (exacerbation-free, OCS-free, asthma control test ≥ 20, and FEV1 ≥ 80% of predicted) [5], and the “Thomas” criteria are equal and also use this lung function cutoff; therefore, this will be considered as a unique definition for this study [6]. The SANI criteria propose a differentiation between partial and complete clinical remission in patients needing no further OCS therapy and depend on the presence of two or all of the following: exacerbations, absence of asthma symptoms, and stabilization of lung function [7]. The primary distinction among the definitions relates to the lung function. Rather than an 80% FEV1, Lommatzsch et al. [8] and the SANI propose the “stabilization” of the lung function, as do the consensus criteria by Menzies-Gow et al. [9]. For our analyses, we considered “stable” to be defined as a consistent FEV1 value measured in liters, allowing for a variability considered “physiological” in lung function test standards (i.e., an improved value or a maximal decline from a previous test of 150 mL) [9].

Exacerbation was defined as a worsening disease requiring the use of systemic steroids or, for chronic steroid users, a temporary increase in the daily dose, for at least 3 days. Hospitalizations were described independently by exacerbations.

The steroid dose was calculated in two ways: first, as a cumulative dose measured in g/year, to support the precise consideration of the steroid-sparing effects of the drug in steroid-dependent and steroid-independent populations, and second, as an average, measured in milligrams, in steroid-dependent patients.

### 2.4. Statistical Analysis

The means and standard deviations (SDs) were used to describe continuous variables. Categorical variables were presented as counts and percentage distributions across different categories. The data were analyzed using an analysis of variance, student’s *t*-test, Fisher’s exact test, univariate analysis, one-sample *t*-tests, and one-proportion z-tests, as well as multiple regression as applicable. A *p* value was considered significant at ≤0.05.

## 3. Results

### 3.1. Patients and Definitions Applied

**Figure 1:**
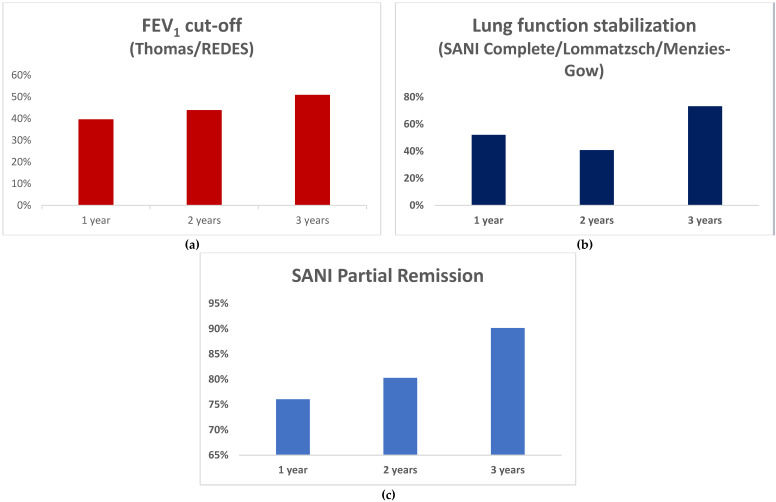
Percentages of patients during 3 years of observation, according to (**a**) FEV1 cutoff; (**b**) lung function stabilization (SANI complete remission/Lommatzsch/Menzies-Gow); (**c**) SANI partial remission.

The study included data from 71 patients (51% male), with a mean age of 59 ± 12 years and a mean age at disease onset of 43 ± 16 years. The baseline data are summarized in Table 1. Using the criteria suggested by REDES and Thomas et al., we classified the same patients as being in remission. With the application of the criteria by Lommatzsch et al. [8], the SANI (complete remission), and Menzies-Gow et al. [9], the results also overlapped, differing from the other two sources in defining remission in terms of the respiratory function. For clarity, given the overlap, we will refer to ‘‘FEV1 cut-off’ to indicate cases meeting REDES and Thomas criteria and to “lung function stabilization” to indicate cases meeting criteria from the other three sources (Lommatzsch et al. [8], SANI complete remission, and Menzies-Gow et al. [9]). We focused the analysis on the most stringent of these criteria (SANI complete and REDES), but in Figure 1 and Table 2, we present remission trends during the 3 years based on each set of remission criteria, including the SANI partial remission criteria.

### 3.2. One-Year Remission

With remission defined using the “FEV1 cutoff” definition (Table 3), at 1 year, we observed a difference in the patients experiencing remission and those not reaching it based on their body mass index (BMI), with a lower BMI linked to a better response (24.6 ± 2.7 vs. 26.3 ± 3.7 kg/m^2^; *p* = 0.05). Also, steroid dependence was lower for patients experiencing remission at 12 months compared with those who were not (25% vs. 50%; *p* = 0.028). Lung function tests were better at the baseline among those achieving remission at 1 year compared with those who did not (FEV1%: 85 ± 25 vs. 68 ± 23; *p* < 0.0001).

With the classification according to “lung function stabilization” (Table 3), we found differences in the baseline exacerbations (3.14 ± 1.89 vs. 4.29 ± 2.87; *p* = 0.046) and steroid dependence, which emerged as an unfavorable factor for remission in the first year (OCS-dependent: 22% vs. 62%; *p* = 0.0006), as well as daily steroid dose (2.13 ± 3.55 vs. 4.33 ± 3.70 g/y; *p* = 0.013). Regarding biomarkers, patients in remission at 1 year had lower FeNO at baseline (46 ± 19 vs. 71 ± 54; *p* = 0.011).

### 3.3. Long-Term Remission

At 3 years, using the “FEV1 cut-off” classification (Table 3, Figure 1a), the remission and non-remission groups did not differ at baseline by sex (58% vs. 41% male; *p* = 0.192), age (58 vs. 61 years; *p* = 0.244), years of disease (41 vs. 46 years; *p* = 0.181), or number of exacerbations (3.14 vs. 4.26; *p* = 0.054), even though having fewer exacerbations had been associated with an increased tendency to experience remission as early as the first year. The groups did not differ significantly by the systemic steroid dose (3.30 vs. 3.06 g/y; *p* = 0.794), steroid dependence (36% vs. 44%; *p* = 0.471), eosinophils (587 vs. 776; *p* = 0.229), or FeNO (56 vs. 60; *p* = 0.640). Chronic rhinosinusitis with nasal polyps at baseline (CRSwNP) was also not predictive of remission (56% vs. 53%; *p* = 0.914). The lung function (FEV1), however, was higher at baseline in patients with remission at 3 years (FEV1%: 85% vs. 62%; *p* < 0.0001), and the baseline BMI was slightly lower in those in complete remission at 3 years (24.1 vs. 26.6 kg/m^2^; *p* = 0.0186).

**Figure 2:**
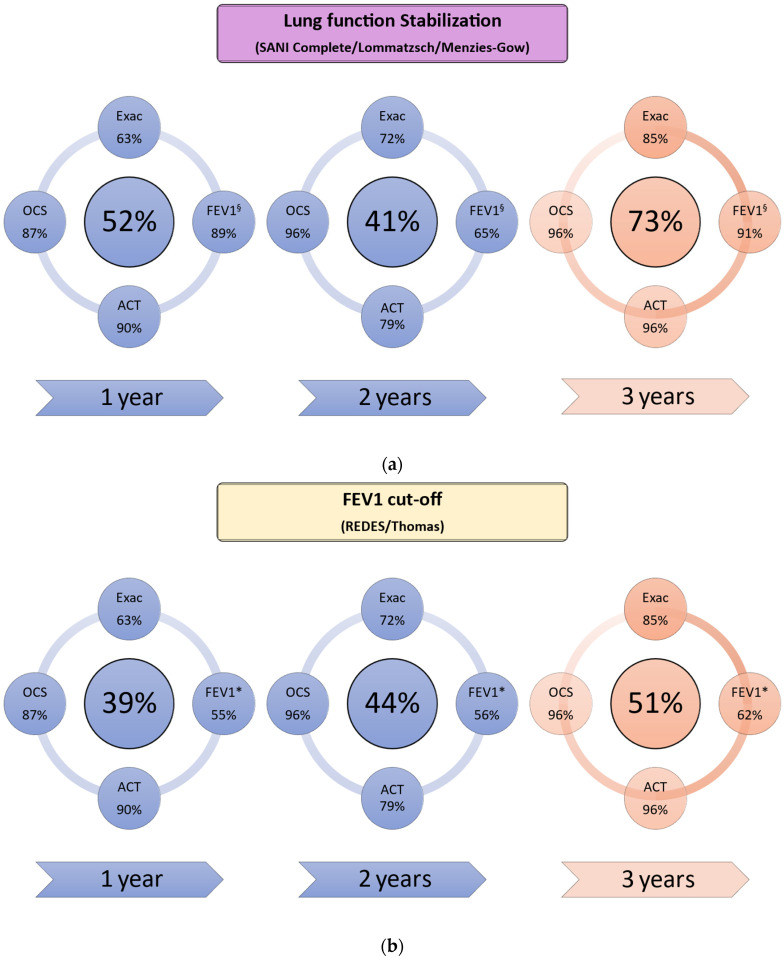
Patients experiencing remission with the different definitions ((**a**) lung function stabilization and (**b**) FEV1 cutoff), with a subanalysis regarding the various criteria by year. *—FEV1 ≥ 80%; ^§^—FEV1 “improved or stable”.

The associations at 1 year with the baseline factors among patients classified according to “lung function stabilization” did not persist at 3 years. We found no differences at 3 years between patients who were in remission compared with those who were not (Figure 1b and Figure 2). The proportion of patients in partial remission at 1 year, classified according to the SANI partial remission criteria, increased from 76% at 1 year to 90% at 3 years. Among the patients classified as having experienced partial remission at year 1, 22% were classified as having reached complete remission at 3 years.

By analyzing the remission trends over the three years using the FEV1 classification, we determined that 32% of patients maintained remission throughout the observation period, while using classifications based on the stabilization of function, we observed maintenance in 21% of the observed cohort.

### 3.4. Predictors of Clinical Remission with ‘FEV1 Cutoff’ Criteria

The odds ratios (ORs) were calculated for clinical and functional variables related to remission at year 1. The univariate analysis revealed that only the FEV1 (OR, 2.43; 95% confidence interval [CI], 1.30–4.51; *p* < 0.005) was an independent predictor of first-year remission. A regression analysis of the clinical and functional data related to remission at year 3 confirmed the persistence of FEV1 as a predictor of remission (OR, 3.72; 95% CI, 1.71–8.07; *p* < 0.001) and identified a higher number of eosinophils (OR, 1.03; 95% CI, 1.01–1.05; *p* < 0.008) as a favorable indicator of remission at 3 years. In contrast, a higher BMI (OR, 0.83; 95% CI, 0.71–0.98; *p* < 0.03) was associated with a reduced likelihood of remission.

### 3.5. Predictors of Clinical Remission with ‘Lung Function Stabilization’ Criteria

The factors at the first year influencing remission, classified using the SANI definition, were the FeNO (OR, 0.97; 95% CI, 0.95–1.00; *p* = 0.02) and OCS values (OR, 0.82; 95% CI, 0.98–2.73; *p* = 0.03). At 3 years, however, no factor was a predictor of remission.

## 4. Discussion

The concept of clinical remission encompasses a comprehensive and holistic approach to evaluating drug efficacy and effectiveness. It combines multiple parameters, rather than the single endpoints generally used in clinical trials, and facilitates more comprehensive conclusions about a treatment [10]. Here, we found that clinical remission is possible for patients with severe asthma treated with mepolizumab.

Remission has often been associated with disease modification, as is seen with rheumatoid arthritis or inflammatory bowel disease, but this concept has not become as associated with asthma or with the evaluation of approved drugs for the condition [8,11]. Lommatzsch et al. proposed defining a disease-modifying anti-asthmatic drug as “any drug class that can potentially achieve the goal of asthma remission”. We have reported that the effects of allergen-specific immunotherapy meet this description [12]. In addition, based on the initial promising data for biologicals, we assessed this treatment as a disease-modifying therapy [11,13]. The current findings offer further support for the ability of biologics to lead to remission.

Our analysis demonstrated the disease-modifying effect of mepolizumab, with a substantial proportion of patients in our selected sample experiencing clinical remission. We believe that these findings present a starting point for evaluating mepolizumab as a disease-modifying drug.

A key finding is the progressive increase in patients experiencing remission from year 1 to year 3. This result implies that patients who were not in remission after a year should be assessed with caution before any change in their treatment, as a longer treatment might yield a remission-related benefit. Depending on the criteria used for classifying remission, remission rates ranged from 39% to 52% at 1 year but increased by year 3 to 51%–73%, with a slight dip in between when using the SANI definition. These results are in accordance with data from extension trials [14] and real-world studies [4,15], showing an improved effectiveness over time with mepolizumab in terms of reduced exacerbations, steroid use, and better disease control.

Another important finding to highlight is the baseline characteristics of patients who experienced remission, which shows some agreement with findings from the REDES study [5]. Both studies identified baseline steroid dependence as a limiting factor in remission with mepolizumab at 1 year. The extension of the treatment time to 3 years in our study showed that by the second year, steroid dependence was no longer a determining factor in remission, leading us to conclude that steroid dependence slowed rather than prevented remission. When remission was defined using the SANI criteria, steroid dependence and the mean daily dose of the OCS were inhibitory factors in remission at the end of year 1 but lost their effect in the later years. All classifications include the complete discontinuation of steroids as a criterion for remission. Patients on a high daily dose at baseline might take longer to achieve a permanent discontinuation, past the initial 12-month time point, as already reported from long-term clinical studies [3,15,16].

In contrast to their common criterion of steroid discontinuation, the classifications diverge regarding the inclusion of respiratory function as a criterion for remission. REDES and Thomas incorporate stabilization of the FEV1 at values ≥80%, whereas Lommatzsch and Menzies-Gow et al. [9] position stabilization as sufficient, as the SANI suggests. The use of a fixed cutoff, such as 80%, means that patients whose spirometry is initially in the normal range or near it are likelier to meet the remission definition sooner.

As described in the Methods, for this study, “stable” spirometry was considered as the maximal decrease of 150 mL from the previous value, indicated in the guidelines on spirometry interpretation as a borderline parameter for “physiological” variation between tests [17]. Regarding the “hot point” of respiratory function, further comparisons within the fields of asthma and respiratory pathophysiology are needed.

The BMI appears to have influenced remission in “FEV1 cutoff” classifications in the first year and seems to have persisted as a factor throughout the study period. Weight has been ruled out as diminishing the drug’s effectiveness [18], but it could play a role in the respiratory function and steroid resistance [19]. On average, as the BMI increases, the lung function declines [20,21], with increases in proinflammatory cytokines and other factors produced by adipocytes [22], reduced physiological airway distension, and the deposition of adipocytes in the bronchial walls [23].

Regarding the “lung function stabilization” classifications, higher sinonasal outcome test 22 (SNOT-22) values at baseline appeared to negatively affect the odds of remission after 1 year of treatment. With the longer observation period of 3 years, we found that as with OCS use, a high SNOT-22 seemed to delay remission rather than prevent it entirely. A recent real-world study by the SANI group confirmed the relevance of longer term mepolizumab treatment for nasal symptoms as measured using the SNOT-22, showing that continuing therapy not only maintained the effectiveness but also progressively improved it [15]. A subanalysis of the REALITI-A study further confirmed this pattern, showing that after 1 year of treatment, CRSwNP no longer affected the mepolizumab effectiveness [24].

The SANI definition of remission is the only one of those considered here that introduces the distinction between partial and complete remission, a significant differentiation that allows for a fuller evaluation of drug effects. Although complete remission is the ultimate goal, it is not always possible. An evaluation of the degrees of treatment efficacy rather than a binary outcome of complete remission or not provides undoubted advantages. The concept of partial remission has been used for other conditions, such as diabetes, to describe patients with an incomplete treatment response based on complete remission criteria but with some satisfactory control of symptoms [25]. In the case of asthma, according to the SANI, the definitive criterion for remission, complete or partial, is the discontinuation of the steroid therapy, emphasizing the systemic steroid-sparing effect expected from biologic drugs [26,27]. The current analysis indicates that patients in partial remission see a gradual improvement over time (Figure 1c). With the inclusion of partial remission as a benchmark, clinicians have better information for decisions about continuing or replacing the therapy. Here, 22% of the cases classified as being in partial remission in the first year went on to complete remission in the third year, emphasizing the importance of time in this context.

Compared with previously published findings, our cohort of patients was more likely to be in remission by the end of the study [5,28]. One reason may be that we included only patients who continued the therapy for the entire period and not those who discontinued it for ineffectiveness or other reasons, so that our cohort was predisposed to experience some effect. In their Australian registry study of patients with severe asthma treated with mepolizumab and omalizumab, Thomas et al. [28] reported a 1-year remission rate of 29.3% (or 25.2% when the respiratory function parameter was also considered), as compared with 39% to 52% in our patients at the same time point. To try to clarify possible reasons for this difference, we considered the predisposing factors for remission identified in that study and compared them with the baseline data from our cohort (Table 4). The results highlight some interesting features. In both cases, the BMI was a common factor in remission in the first year of treatment, and the BMI values were significantly higher in the earlier study, possibly explaining our higher remission rates.

We used a univariate analysis to define which clinical or functional variables might be predictive factors for remission in the first and third years. With Gibson’s criteria, the FEV1 was confirmed as a predictor of remission in both the first and third years, as might be expected due to the necessity of reaching and maintaining at least an 80% value; therefore, higher baseline values make it easier for patients to achieve this goal compared to those recording a lower baseline respiratory function. Stability or an improvement in the respiratory function is implied in the definition of remission itself. The functional aspect is always considered in defining remission across the classifications we investigated, although defined differently, either more generally as stabilization/optimization of the respiratory function or as FEV1 ≥ 80% of the predicted value.

In the analysis for the third year, higher eosinophils and a lower BMI were correlated with remission; they were not correlated in the first year. The univariate analysis thus indicated that these two factors are crucial in predicting remission over the long term. In particular, lower remission rates for patients with higher BMIs implies that these patients experienced less symptom control and more frequently exhibited the non-eosinophilic phenotype, which is associated with greater systemic oxidative stress that can reduce the response to therapy and prevent remission.

The univariate analysis also highlighted the role of steroids in remission when the SANI criteria were applied. Patients using a higher dose in the year before initiating the biologic therapy had lower odds of remission at year 1. The results were similar with FeNO, with high values linked to lower odds of remission in the first year. With the longer observation at 3 years, however, these factors were no longer significant, again emphasizing that they may increase the time to remission but not prevent it (Table 4 and Table A2).

## 5. Limitations

The size of the sample, despite being the first in real life, is small. All included patients showed at least one positive effect from mepolizumab administration in the first year of treatment, and thus continued treatment for the next 2 years, which is a potential limitation on the accurate calculation of remission rates. The values likely would be more precise if patients who experienced a poor response and discontinued the treatment before the 3-year time point were also included. Furthermore, all patients were treated monthly in the hospital, indicating a good adherence to the inhaled and biological therapies, which would probably increase the likelihood of better disease control. An additional limitation is that the anamnestic and functional data were collected annually rather than every 6 months.

## 6. Conclusions

In conclusion, the results highlight the possibility of clinical remission in patients with severe asthma treated with mepolizumab, with remission assessed using six published definitions. The findings suggest that the length of treatment is important, as many patients not in remission at 1 year were in remission after continuing therapy longer. In addition, the respiratory function is important as a criterion. Finally, introducing biologics early in the disease course could be a factor influencing the effectiveness of the therapy in the context of remission.

## Figures and Tables

**Table 1:** Baseline characteristics of patients.

*n* = 71	Baseline
Male, *n* (%)	36 (51)
Female, *n* (%)	35 (49)
Age (year), mean (±SD)	59 (12)
Age onset (year), mean (±SD)	43 (16)
<18 year	4 (6)
18–40 year	20 (28)
40–65 year	40 (56)
>65 year	7 (10)
Asthma duration (year), mean (±SD)	16.1 (11.3)
Smokers, *n* (%)	4 (6)
BMI (kg/m^2^), mean (±SD)	25.7 (3.4)
OCS dependent, *n* %)	29 (41)
CRSwNP, *n* (%)	39 (55)
Exacerbation ^§^, mean (±SD)	3.7 (2.4)
Hospitalization, *n* (%)	14 (20)
OCS dose ^+^ (dependent), mean (±SD)	15.6 (10.5)
OCS dose (g/year), mean (±SD)	3.2 (3.7)
FEV1 (L), mean (±SD)	2.18 (0.9)
FEV1 %, mean (±SD)	75 (25)
Eosinophils ^#^, mean (±SD)	677 (643)
FeNO (ppb), mean (±SD)	58 (41)
ACT, mean (±SD)	18 (3)
SNOT-22, mean (±SD)	51 (15)

Exacerbation rate (^§^); grams of prednisone equivalent (^+^); geometric mean expressed in cells/µL (^#^); standard deviation (SD); body mass index (BMI); oral corticosteroids (OCS); chronic rhinosinusitis with nasal polyps (CRSwNP); forced expiratory volume in the first second (FEV1); fractional exhaled nitric oxide (FeNO); asthma control test (ACT); sinonasal outcome test 22 (SNOT-22).

**Table 2:** Percentage and absolute number of patients in clinical remission, according to FEV1 cutoff definitions (REDES 4 and Thomas), SANI partial remission, and lung function stabilization criteria (SANI complete remission/Lommatzsch/Menzies-Gow).

	FEV1 Cutoff (Redes–Thomas)	SANI Partial	Lung Function Stabilization (SANI Complete/Lommatzsch/Menzies-Gow)
1 year	39% (28)	76% (54)	52% (37)
2 year	44% (31)	80% (57)	41% (29)
3 year	51% (36)	90% (64)	73% (52)

Data are expressed as percentage and (absolute number).

**Table 3:** Baseline characteristics of patients in remission at 1 and 3 years according to (**a**) the criteria of FEV1 cutoff (REDES/Thomas); (**b**) lung function stabilization (SANI complete remission/Lommatzsch/Menzies-Gow).

**FEV1 Cutoff (REDES/Thomas)**						
**(a)**	**1 Year**			**3 Years**		
	**Remission**	**No Remission**		**Remission**	**No Remission**	
			***p*-Value**			***p*-Value**
N (%)	28 (39%)	43 (61%)		36 (51%)	35 (49%)	
Male, n (%)	11 (39%)	25 (58%)	0.12	21 (58%)	15 (42%)	0.192
Age (year), mean (±SD)	58 (13)	60 (11)	0.442	58 (13)	61 (10)	0.244
Age at onset (year), mean (±SD)	41 (17)	46 (16)	0.265	41 (17)	46 (16)	0.181
Smoker, n (%)	3 (11%)	1 (2%)	0.134	4 (0.06)	0 (0)	0.04
BMI (kg/m^2^)	24.6 (2.7)	26.3 (3.7)	0.05	24.7 (2.8)	26.6 (3.7)	0.0186
Exacerbation ^§^, mean (±SD)	3.21 (1.97)	4.0 (2.71)	0.19	3.14 (2.1)	4.26 (2.69)	0.054
Hospitalizations, mean (±SD)	0.11 (0.31)	0.33 (0.61)	0.084	0.22 (0.54)	0.26 (0.51)	0.779
OCS dose (g/year) ^+^, mean (±SD)	2.42 (3.97)	3.67 (3.58)	0.172	3.3 (4.61)	3.06 (2.68)	0.794
OCS dependent, n (%)	7 (25%)	22 (51%)	0.028	13 (36%)	16 (44%)	0.471
CRSwNP, n (%)	14 (50%)	25 (58%)	0.501	20 (56%)	19 (53%)	0.914
FEV1 (L), mean (±SD)	2.50 (0.98)	1.98 (0.80)	0.016	2.55 (0.92)	1.81 (0.72)	0.0003
FEV1 %, mean (±SD)	85 (25)	68 (23)	<0.0001	85 (27)	64 (18)	<0.0001
Eosinophils ^#^, mean (±SD)	602 (593)	729 (685)	0.428	587 (467)	776 (796)	0.229
FeNO ^ç^, mean (±SD)	55 (51)	60 (34)	0.674	56 (46)	60 (36)	0.64
ACT	18 (3)	18 (4)	0.472	19 (3)	17 (3)	0.163
SNOT-22	43 (19)	50 (15)	0.153	46 (18)	49 (16)	0.568
**Lung Function Stabilization (SANI Complete Remission/Lommatzsch/Menzies-Gow)**						
**(b)**	**1 Year**			**3 Years**		
	**Remission**	**No Remission**		**Remission**	**No Remission**	
			***p*-Value**			***p*-Value**
N (%)	**37 (52%)**	34 (48%)		**52 (73%)**	19 (27%)	0.051
Male, n (%)	18 (49%)	18 (53%)	0.718	30 (58%)	6 (32%)	0.651
Age (year), mean (±SD)	59 (13)	60 (10)	0.742	60 (12)	59 (10)	0.467
Age at onset (year), mean (±SD)	43 (16)	44 (17)	0.84	43 (17)	46 (16)	0.213
Smoker, n (%)	4 (11%)	0 (0)	0.05	4 (8%)	0 (0)	0.939
BMI (kg/m^2^)	25.3 (3.5)	26.1 (3.3)	0.295	25.7 (3.5)	25.6 (3.3)	0.238
Exacerbation ^§^, mean (±SD)	3.14 (1.82)	4.29 (2.87)	0.046	3.48 (2.2)	4.26 (3.05)	0.818
Hospitalizations, mean (±SD)	0.11 (0.31)	0.38 (0.65)	0.025	0.23 (0.51)	0.26 (0.56)	0.482
OCS dose (g/year) ^+^, mean (±SD)	2.13 (3.55)	4.33 (3.70)	0.013	2.99 (4.06)	3.71 (2.84)	0.222
OCS dependent, n (%)	8 (22%)	21 (62%)	0.0006	19 (37%)	10 (53%)	0.206
CRSwNP, n (%)	19 (51%)	20 (59%)	0.527	22 (42%)	13 (68%)	0.476
FEV1 (L), mean (±SD)	2.19 (0.88)	2.17 (0.94)	0.924	2.23 (0.96)	2.06 (0.74)	0.913
FEV1 %, mean (±SD)	73 (25)	76 (26)	0.961	76 (24)	72 (27)	0.102
Eosinophils ^#^, mean (±SD)	705 (788)	647 (459)	0.711	602 (561)	892 (828)	0.237
FeNO ^ç^, mean (±SD)	46 (19)	71 (54)	0.011	54 (45)	68 (30)	0.745
ACT	18 (3)	18 (4)	0.519	18 (4)	18 (3)	0.127
SNOT-22	40 (19)	55 (8)	0.002	45 (19)	53 (53)	0.051

Exacerbations rate (^§^); grams of prednisone equivalent (^+^); geometric mean expressed in cells/µL (^#^); standard deviation (SD); body mass index (BMI); oral corticosteroids (OCS); chronic rhinosinusitis with nasal polyps (CRSwNP); forced expiratory volume in the first second (FEV1); fractional exhaled nitric oxide (FeNO); asthma control test (ACT); sinonasal outcome test 22 (SNOT-22); ppb (^ç^).

**Table 4:** Univariate analysis of remission at 1 and 3 years according to REDES/Gibson and SANI complete remission/Lommatzsch/Menzies-Gow definitions.

FEV1 Cutoff (Gibson/Thomas)				Lung Function Stabilization (SANI Complete Remission/Lommatzch/Menzies-Gow)			
**1 year**	**OR**	**CI**	***p***	**1 year**	**OR**	**CI**	***p***
Sex (M vs. F)	0.47	0.18–1.23	0.12	Sex (M vs. F)	0.84	0.33–2.14	0.72
Age, year	0.98	0.95–1.03	0.45	Age, year	0.99	0.95–1.03	0.74
Age at onset, year	0.98	0.95–1.01	0.26	Age at onset, year	1.00	0.97–1.03	0.84
BMI (kg/m^2^)	0.85	0.85–0.72	0.06	BMI (kg/m^2^)	0.93	0.81–1.07	0.32
FEV1 (L)	2.43	1.30–4.51	0.005	FEV1 (L)	1.03	0.61–1.72	0.92
FeNO (ppb)	1.00	0.98–1.02	0.89	FeNO (ppb)	0.97	0.95–1.00	0.02
Eosinophils (cells/µL)	1.00	0.99–1.02	0.55	Eosinophils (cells/µL)	1.00	0.99–1.00	0.71
OCS (g/year)	0.001	0.00–inf	0.99	OCS (g/year)	0.82	0.98–2.73	0.03
Nasal polyps (year/n)	0.72	0.28–1.87	0.50	Nasal polyps (year/n)	0.53	0.29–1.89	0.74
**3 year**	**OR**	**CI**	***p***	**3 year**	**OR**	**CI**	***p***
Sex (M vs. F)	1.87	0.73–4.79	0.19	Sex (M vs. F)	2.95	0.97–8.99	0.06
Age, year	0.98	0.94–1.02	0.26	Age, year	1.01	0.97–1.06	0.68
Age at onset, year	0.98	0.95–1.01	0.18	Age at onset, year	0.99	0.96–1.02	0.46
BMI (kg/m^2^)	0.83	0.71–0.98	0.03	BMI (kg/m^2^)	1.01	0.86–1.18	0.93
FEV1 (L)	3.72	1.71–8.07	0.001	FEV1 (L)	1.25	0.68–2.31	0.47
FeNO (ppb)	1.01	0.99–1.04	0.386	FeNO (ppb)	0.99	0.98–1.01	0.26
Eosinophils (cells/µL)	1.03	1.01–1.05	0.008	Eosinophils (cells/µL)	0.99	0.99–1.00	0.13
OCS (g/year)	1.37	0.49–3.87	0.55	OCS (g/year)	0.46	0.15–1.40	0.17
OCS 3 year (dose prednisone)	0.002	0.00–inf	0.99	OCS 3 year (dose prednisone)	0.99	0.94–1.04	0.60

CI, confidence interval; OR, odds ratio.

## Data Availability

Data are contained within the article.

## Appendix A

**Table A1:** Comparison between the cohort of Thomas et al. [28] and our patients.

	Thomas (N = 278)	Current Cohort (N = 71)	*p*
Age (year), mean (±SD)	59.41	59 (12)	0.771
Male, n (%)	114 (41)	36 (51)	0.086
BMI (kg/m^2^), mean (±SD)	29.7	25.7 (3.4)	<0.0001
Duration of disease (year), mean (±SD)	29.51	16.1 (11.3)	0.0001
Hospitalization, n (%)	76 (27)	14 (20)	0.184
OCS dependent, n (%)	118 (42.4)	29 (41)	0.733
OCS in OCS user	10	15	0.089
FEV1%, mean (±SD)	62	75 (25)	<0.0001

**Table A2:** Predictors of remission according to FEV1 cutoff (REDES/Gibson) and lung function stabilization (SANI complete remission/Lommatzsch/Menzies-Gow) criteria.

Predictive Factors of Remission		
	1st Year	3rd Year
FEV1 cutoff (REDES/Thomas)	↑ FEV1	↑ FEV1 ↑ Eosinophils ↓ Lower BMI
Lung function stabilization (SANI complete remission/Lommatzsch/Menzies-Gow)	↑ FeNO ↓ OCS g/year	None

Legend: ↑ means highest level of a single parameters, ↓ means a lower level of a parameters.
